# Introduction of Avian metapneumovirus subtype A to the United States: molecular insights and implications

**DOI:** 10.3389/fmicb.2024.1428248

**Published:** 2024-07-05

**Authors:** Iryna V. Goraichuk, Mia K. Torchetti, Mary L. Killian, Darrell R. Kapczynski, Kathleen Sary, Arun Kulkarni, David L. Suarez

**Affiliations:** ^1^Southeast Poultry Research Laboratory, U.S. National Poultry Research Center, Agricultural Research Service, U.S. Department of Agriculture, Athens, GA, United States; ^2^National Veterinary Services Laboratories, Animal and Plant Health Inspection Service, U.S. Department of Agriculture, Ames, IA, United States; ^3^Georgia Poultry Laboratory Network, Gainesville, GA, United States

**Keywords:** aMPV-A, Avian metapneumovirus, NGS, next-generation sequencing, Illumina, whole-genome sequencing, RT-qPCR, subtypes

## Abstract

Avian metapneumovirus (aMPV) poses a significant threat to the poultry industry worldwide, primarily affecting turkeys and chickens. The recent detection of aMPV-A and -B subtypes in the United States marks a significant shift after a prolonged period free of aMPV following the eradication of the previously circulating subtype C. Hence, the demand for molecular diagnostic tests for aMPV has arisen due to their limited availability in the US market. In this study, we present the molecular characterization based on the complete genome sequence of aMPV subtype A, which was detected in the US for the first time. Four RT-qPCR positive samples were subjected to next-generation sequencing analysis, resulting in the assembly of one complete and one near-complete genome sequences. Phylogenetic analysis revealed that the isolated strains clustered within the aMPV-A subtype and were most closely related to recent Mexican strains. A detailed amino acid analysis identified unique mutations in the G gene of the US isolates compared to Mexican strains. Additionally, we compared the performance, cross-reactivity, and limit of detection of our revised aMPV subtype-specific RT-qPCR test with two commercial kits, demonstrating similar detection and subtyping capabilities. These findings highlight the importance of accurate diagnostic methods for disease management in the poultry industry, provide valuable insights into the epidemiology of aMPV, and underscore the need for continued vigilance and surveillance to mitigate its impact on poultry production.

## 1 Introduction

Avian metapneumovirus (aMPV), a member of the genus *Metapneumovirus* in the family *Pneumoviridae*, poses a significant threat to the poultry industry worldwide, predominantly impacting turkeys and chickens (Cook, 2000; Rima et al., 2017; Walker et al., 2019). The disease associated with aMPV infection, commonly known as avian rhinotracheitis or turkey rhinotracheitis, causes respiratory tract infections and decreased egg production, resulting in substantial economic losses (Shin et al., 2002). Co-infection with primary or secondary bacterial pathogens, such as *Escherichia coli* (Turpin et al., 2002), *Mycoplasma gallisepticum* (Naylor et al., 1992), *Ornithobacterium rhinotracheale* (Marien et al., 2005; Zuo et al., 2018), or lentogenic Newcastle disease virus (Turpin et al., 2002), often leads to more severe clinical signs, swollen head syndrome, and high morbidity rates (Giovanardi et al., 2014; Salles et al., 2023). While turkeys, chickens, and ducks are the primary host species for aMPVs, these viruses have also been detected in various wild bird species (Brown et al., 2019).

Currently, aMPV is classified into four subtypes (aMPV-A, -B, -C, and -D) using various different techniques, including reactivity against monoclonal antibodies, cross-reactivity in the enzyme-linked immunosorbent assay (ELISA), neutralization test, and nucleotide sequence analysis (WOAH, 2022). Neutralization tests based on monoclonal antibodies to the spike glycoprotein G have shown that while subtypes A and B belong to a single serotype, subtype C represents a second serotype of aMPV (WOAH, 2022). The first detection of aMPV occurred in turkeys with respiratory disease in South Africa in 1978 (Buys and Du Preez, 1980; Salles et al., 2023), followed by its detection in England during the mid-1980s. It then rapidly spread across Europe (Anon, 1985; Giraud et al., 1986; Hafez and Woernle, 1989; Cook and Cavanagh, 1993) and was later detected on other continents, except for Australia, where it has never been detected (Jones, 1996; Canuti et al., 2019). Early aMPV isolates circulating in Europe during the 1980s were later classified based on genetic and antigenic differences into two distinct subtypes: aMPV-A (formed by strains CVL 14/1 from the UK and 1,556 from France) and aMPV-B (formed by the other continental European strains 2,119 from Italy, 657/4 from Humgary, and 872S from Spain) (Juhasz and Easton, 1994; Rautenschlein et al., 2013). Retrospective analyses revealed that the first identified strains in South Africa in 1978 and the UK in 1985 were of subtype A (Cook et al., 1993). Later, a third subtype aMPV-C was first identified in turkeys in the United States in 1996 (Seal, 1998) and a related C sublineage was reported in a Muscovy duck (*Cairina moschata*) in France in 1999 (Toquin et al., 1999, 2006). Additionally, a retrospective study conducted on archival samples from turkeys in France in 1985 (Bäyon-Auboyer et al., 2000) revealed the presence of a fourth distinct subtype, designated as aMPV-D, which has not been reported since. Recently, two new unclassified subtypes were discovered in a Monk parakeet (*Myiopsitta monachus*) (Retallack et al., 2019) and a great black-backed gull (*Larus marinus*) (Canuti et al., 2019), tentatively increasing the number of known aMPV subtypes to six.

Presently, aMPVs are found worldwide, primarily in regions with poultry production or along migratory bird routes. Subtypes A and B are prevalent in Europe (Andreopoulou et al., 2019; Franzo et al., 2020; Goraichuk et al., 2020; Mescolini et al., 2021), Brazil (Felippe et al., 2011; Rizotto et al., 2017; Kariithi et al., 2023), and African countries (Tegegne et al., 2020), with aMPV-A becoming less frequently detected and aMPV-B being the most prevalent subtype (Graziosi et al., 2022). Subtype C has been identified in various countries, including the United States (Seal, 2000; Shin et al., 2002), Canada (Jardine et al., 2018), China (Wei et al., 2013; Sun et al., 2014), South Korea (Lee et al., 2007), France (Toquin et al., 2006), Netherlands (Jesse et al., 2022), and Italy (Legnardi et al., 2021; Graziosi et al., 2022; Tucciarone et al., 2022). Increasing evidence suggests that wild birds may play a significant role as carriers in the spread of the virus, though their contribution to virus transmission to domestic poultry flocks remains unclear (Graziosi et al., 2022; Jesse et al., 2022; Kariithi et al., 2022).

The United States was considered free of aMPV until the detection of a new aMPV-C subtype in Colorado in 1996 (Cook et al., 1999). This subtype later became endemic in turkey flocks and was subsequently reported in multiple states across the West (Colorado, Minnesota, North Dakota, South Dakota, Iowa, Ohio, and Wisconsin) and wild birds in the Southeast (Georgia, South Carolina, and Arkansas) regions in the late 1990s and early 2000s before vaccination was initiated and the virus was eradicated (Goyal et al., 2000; Bennett et al., 2004; Turpin et al., 2008). Following the implementation of live attenuated vaccines alongside biosecurity measures in endemic areas, the incidence of aMPV-C outbreaks notably decreased. Due to the success of these measures which led to a lack of virus detection in poultry or wildlife, the country maintained an aMPV-free status for over a decade. However, recently National Veterinary Services Laboratory and the National Animal Health Laboratory Network (NAHLN) confirmed the detection of aMPV-B in turkeys and broilers in West Virginia, Virginia, Georgia, South Carolina, and North Carolina, with the following detection of aMPV-A in Texas and California, which pose renewed challenges (Luqman et al., 2024; UGA, 2024; United Egg Producers, 2024).

The economic significance of aMPV in the poultry industry cannot be overstated, as outbreaks of aMPV-A and -B can result in significant financial losses, including decreased productivity, increased mortality rates, and expenses associated with disease management and control. The global trade of poultry and poultry products necessitates surveillance and rapid detection methods to prevent the spread of aMPV to unaffected regions. Various diagnostic methods, including virus isolation, serological assays (ELISA) (Xu et al., 2021), and molecular techniques (conventional, droplet, or real-time reverse-transcriptase polymerase chain reaction (RT-PCR/ RT-qPCR)), have been employed for the detection of aMPV (Guionie et al., 2007; van Boheemen et al., 2012; Lemaitre et al., 2022; Mo et al., 2022; Wang et al., 2022). PCR assays, in particular, became essential tools due to their sensitivity, specificity, and rapid turnaround time. Given the genetic variability of aMPV subtypes and the emergence of novel strains, subtype-specific PCR tests must be tailored to detect currently circulating strains effectively.

In this study, we report the molecular characterization based on the complete genome of aMPV-A detected for the first time in the United States. Additionally, we optimized an aMPV subtype-specific RT-qPCR test for improved specificity and sensitivity to currently circulating strains. We also compared our optimized test with two commercial aMPV RT-qPCR kits to evaluate it as an alternative while available diagnostic products undergo license and import permits from the Center for Veterinary Biologics (CVB). By elucidating the genetic diversity of aMPVs and assessing the performance of molecular diagnostic assays, we aim to contribute to the ongoing efforts to mitigate the impact of this pathogen on poultry health and welfare of the United States.

## 2 Materials and methods

### 2.1 aMPV RT-qPCR screening

A total of 42 respiratory swab pools and tissue samples from turkey and chicken flocks from California were sent to the NVSL as part of an ongoing aMPV surveillance program among poultry birds. The swab samples were pooled with a maximum of 5 samples per pool according to their collection location (Fresno, Madera, Merced, Sacramento, San Joaquin, and Stanislaus County) at the collection site. Total RNA was extracted from pooled swabs and tissue samples using the MagMax CORE Nucleic Acid Purification Kit (Applied Biosystems, US), following the manufacturer’s recommended protocol. A total of 21 swab pools and 21 tissue samples were screened for aMPV detection and subtyping using multiplex RT-qPCR for A, B, and C subtypes, as described below.

### 2.2 Next-generation sequencing

Four RNA samples were sent to the Southeast Poultry Research Laboratory (SEPRL) for deep NGS analysis. To enhance the yield of viral reads, 12 μL of RNA samples were treated to selectively deplete host-specific (18S, 28S, and mitochondrial) and selected bacterial (16S and 23S) rRNAs using our RNaseH rRNA depletion protocol (Parris et al., 2022; Bakre et al., 2023; Goraichuk et al., 2024). For viral enrichment, sequence-independent, single-primer amplification (SISPA) (Chrzastek et al., 2017) was employed. Briefly, cDNAs were synthesized from 10 μL of the RNaseH-treated RNA using random K-8N primer with SuperScript IV First-Strand Synthesis Kit (Invitrogen, US) and Klenow polymerase (NEB Inc., US). Following Agencourt AMPure XP bead purification (Beckman Coulter Life Sciences, US), 5 μL of the cDNAs were subject to amplification using the Phusion High-Fidelity PCR Kit (NEB Inc., US). After the SISPA amplification step, amplicons were bead purified with a 1:1.8 sample volume to bead volume ratio and used to prepare sequencing libraries with the Nextera DNA Library Preparation Kit (Illumina, US). After quantification of concentrations and average fragment sizes using the Qubit 1X dsDNA High Sensitivity Assay Kit (Thermo-Fisher Scientific, US) and Agilent High Sensitivity D5000 ScreenTape Assay (Agilent Technologies, CA), respectively, the libraries were pooled (4 nM, 10 μL each). Subsequently, the pooled libraries were subject to digestion with 0.2 N NaOH (5 min incubation at room temperature). Following the addition of a control library (5% PhiX library v3) to the diluted library pools (12 pM final concentration), paired-end sequencing (2 × 300 bp) was conducted using the 600-cycle MiSeq Reagent Kit v3 (Illumina, US) on an Illumina MiSeq instrument.

### 2.3 Sequencing assembly and analysis

The Illumina raw sequencing data generated at SEPRL was processed within the Galaxy platform, as described previously (Dimitrov et al., 2017). Quality assessment of raw sequence reads was conducted using FastQC v0.63 (Andrews, 2010), with subsequent removal of residual adaptor sequence and low-quality bases by using Cutadapt v1.16.6 (Martin, 2011). Host reads (*Meleagris gallopavo*) were eliminated using the Burrows-Wheeler Alignment tool (BWA-MEM) (Li and Durbin, 2009), while forward and reverse reads were synchronized and overlapping read pairs were merged using in-house sync_reads v.0.02 tool (Volkening, 2023) and PEAR v.0.9.6.1 (Zhang et al., 2014; Volkening, 2023), respectively. Digital normalization via median k-mer abundance was carried out using the khmer v1.1–1 package (cutoff, 100; k-mer size, 20) (Crusoe et al., 2015). The remaining unmapped reads were subject to *de novo* assembly utilizing MIRA3 v0.0.1 (Chevreux et al., 1999). The aMPV genome scaffold was constructed from the *de novo*-generated contigs, with final genome consensus calling performed using the bam2consensus tool (Volkening, 2023) (minimum base quality of 10; minimum read depth of 3×) following BWA-MEM mapping of raw synchronized aMPV reads to the genome scaffold for high-coverage samples and to the reference sequence aMPV-A/Mexico/MEX/3155/2022 (GeneBank accession number ON854014.1) (Kariithi et al., 2022) for low-coverage samples.

Genome coverage depth was determined using the SAMtools depth (Danecek et al., 2021) command and visualized using the R package ggplot2 (Wickham, 2016). Open reading frames (ORFs) were predicted, and corresponding genes were annotated and validated by comparison with homologous genes/ORFs and coding sequences (CDS) of aMPVs retrieved from GenBank using Geneious Prime v2023.0.1 software.

Bacterial co-infections were identified through taxonomical classification using Kraken2 v2.0.8 with the PlusPF database (Wood and Salzberg, 2014; Wood et al., 2019) within the Galaxy platform. Kraken2 classified reads were further processed with Bracken v2.5 (Lu et al., 2017) to estimate relative abundance at the family level. Individual Bracken taxonomy tables for each sample were merged using the “combine_bracken_outputs.py” Python script. The merged Bracken data was processed with the R application “bracken_plot” (Vill, 2023) to determine and visualize the top 15 taxa with the greatest median relative abundances.

### 2.4 Phylogenetic analysis

Phylogenetic analysis of sequences obtained in this study was conducted based on complete genome and G gene nucleotide sequences. All available complete and near-complete genome sequences of aMPV subtype A-D and unclassified aMPVs, representing all members of the genus *Metapneumovirus*, (*n* = 49), and all available near-complete G gene sequences of aMPV-A (*n* = 24) were retrieved from NCBI GenBank. Multiple sequence alignment was performed using MAFFT v7 (Katoh and Standley, 2013). Phylogenetic trees were constructed using the Maximum Likelihood method with the best-fitting nucleotide substitution model based on the lowest Bayesian Information Criterion (BIC) and Akaike Information Criterion (AIC) scores using MEGA 7 (Kumar et al., 2016). The best-fit model for each phylogenetic tree is described in the figure legends. Tree construction was performed using a 1,000 bootstrap test. A pneumonia virus of mice J3666 (genus *Orthopneumovirus*) (GenBank accession number NC_006579.1) was used as an outgroup for rooting the tree. Evolutionary distances within and between different clades were calculated based on a total of 1,141 positions in the final dataset of all (*n* = 26) near-complete G gene sequences of aMPV-A after the elimination of positions containing gaps and missing data using MEGA 7.

### 2.5 Subtype-specific aMPV RT-qPCR tests comparison

Considering the recent introduction of aMPV-B (Luqman et al., 2024) and the past history of aMPV-C in the US, we enhanced our previously published SEP aMPV-A RT-qPCR test (Kariithi et al., 2022) to additionally detect and subtype aMPV-B and -C as well. To achieve this, previously published forward and reverse primers were modified based on currently available aMPV sequences to reduce sequence variability, and the probe’s label was changed from tetramethylrhodamine (TAMRA) to fluorescein (FAM) with quencher (BHQ1) fluorescent reporter dye (Table 1) (Guionie et al., 2007; Jardine et al., 2018). Reagents and thermocycling conditions were tested for compatibility with the routinely used protocols at the NVSL. All primers were reconstituted to 20 pmol/μL and probes to 6 pmol/μL of working stock concentrations. The optimal reaction concentrations of primers and probes were selected by testing different volumes (Table 1).

**Table 1:** Sequences of primers and probes used in this study.

Target	Primers	Nucleotide sequence (5′ – 3′)	Recommended amount^**^	Source
aMPV-A (G gene)	aMPV-A F	GGA CAT CGG GAG GAG GTA CA	0.5 μL	Kariithi et al. (2022), Guionie et al. (2007)
	aMPV-A SEP* R	CTG CAC TCC TCT AAC ACT GAC TGT T	0.5 μL	
	aMPV-A SEP probe	FAM-CTG ACC TGC ACA GTC ACT ATT GCA CTC ACT GT-BHQ-1	1 μL	
aMPV-B (G gene)	aMPV-B SEP F	GTC CTC AAG CAA GTC CTC AGA AG	1 μL	Guionie et al. (2007)
	aMPV-B SEP R	CCA CAC ACT GTC GTA ATT TGA CCT G	1 μL	
	aMPV-B probe	FAM- CTG GTG TTA TCA GCC TTA GGC TTG ACG CT -BHQ-1	0.5 μL	
aMPV-C (M gene)	aMPV-C F	GGC CCA ATA CTG AAG GTC AA	1 μL	Jardine et al. (2018)
	aMPV-C R	GCT ACT GAT GCA CTA ACA TCA AAG	1 μL	
	aMPV-C probe	FAM-TTT GGC AAT GCT GAC ATT GCA GCC-BHQ-1	2 μL	

^*^SEP – primers that were modified from the original study; ^**^ - recommended primer/probe amount for 1 reaction in 25 μL RT-qPCR reaction mix with primer’s concentration of 20 pmol/ μL and probe’s concentration of 6 pmol/ μL.

All three subtype-specific RT-qPCR tests were carried out in a 25 μL reaction with 5 μL of sample RNA using AgPath-ID One-Step RT-PCR Reagents (Applied Biosystems, US) according to the manufacturer’s recommendations. PCR thermocycling parameters included reverse transcription at 45°C for 10 min, RT inactivation/initial denaturation at 95°C for 10 min, followed by 40 cycles of amplification with denaturation at 94°C for 10 s, annealing at 57°C for 30 s (optics on), and extension at 72°C for 10 s on the QuantStudio 5 Real-Time System (Applied Biosystems, US).

Currently, the US market lacks certified diagnostic kits for aMPV, as it was free of any aMPVs. While the CVB began accepting license and import permit applications for aMPV diagnostic products, it may take some time for those kits to become available in the US. Therefore, we compared the performance (sensitivity and cross-reactivity) of our SEP aMPV subtype-specific RT-qPCR test, as an alternative, to two European commercial tests: Kit 1 – the VetMax Avian Metapneumovirus Kit (Applied Biosystems, France) for RT-qPCR detection of A, B, and C subtypes and Kit 2 – RealPCR AMPV Subgroup A/B Multiplex RNA Mix with RealPCR RNA Master Mix (IDEXX, France) for simultaneous detection of A and B subtypes. The evaluation was performed using RNA extracted from 36 field and reference aMPV samples of A, B, and C subtypes available at SEPRL. Additionally, 10-fold RNA serial dilutions were prepared for aMPV-A, -B, and -C for the comparison of the limit of detection among the three RT-qPCR tests. The limit of detection was considered the last dilution in which all replicates were positive (Ct < 37°C).

GraphPad Prism 9.3.1 was used for data representation and statistical analysis. Mixed-effects analysis was utilized to compare the relative difference of cycle threshold (Ct) values between SEP, Kit 1, and Kit 2 with aMPVs of different subtypes. For statistical purposes, all swab samples with negative RT-qPCR results were assigned a Ct value of 40. The value of *p* < 0.05 was considered statistically significant.

## 3 Results

### 3.1 aMPV screening of field samples

RT-qPCR screening of 42 respiratory swab pools and tissue samples from turkey and chicken flocks resulted in 28 aMPV-A positive samples with RT-qPCR Ct values ranging from 25.8 to 37.9. These included 15 swab pools and 13 tissue samples. Among these, four samples (Table 2) were selected for further analysis and transferred to SEPRL for NGS.

**Table 2:** Summary of aMPV-A positive samples sequenced at the SEPRL.

Sample pool	Host	Collection date	Collection location	Sample type	RT-qPCR, Ct value
24–003038-001	Turkey, grower type	11-16-2023	Merced County, CA, US	respiratory swab pool	32.7
24–003047-001	Turkey, grower type	11-13-2023	Merced County, CA, US	respiratory swab pool	34.1
24–003048-001	Turkey, grower type	12-07-2023	Stanislaus County, CA, US	respiratory swab pool	30.5
24–003049-001	Turkey, grower type	11-28-2023	Merced County, CA, US	respiratory swab pool	27.6

### 3.2 NGS analysis

**Figure 1:**
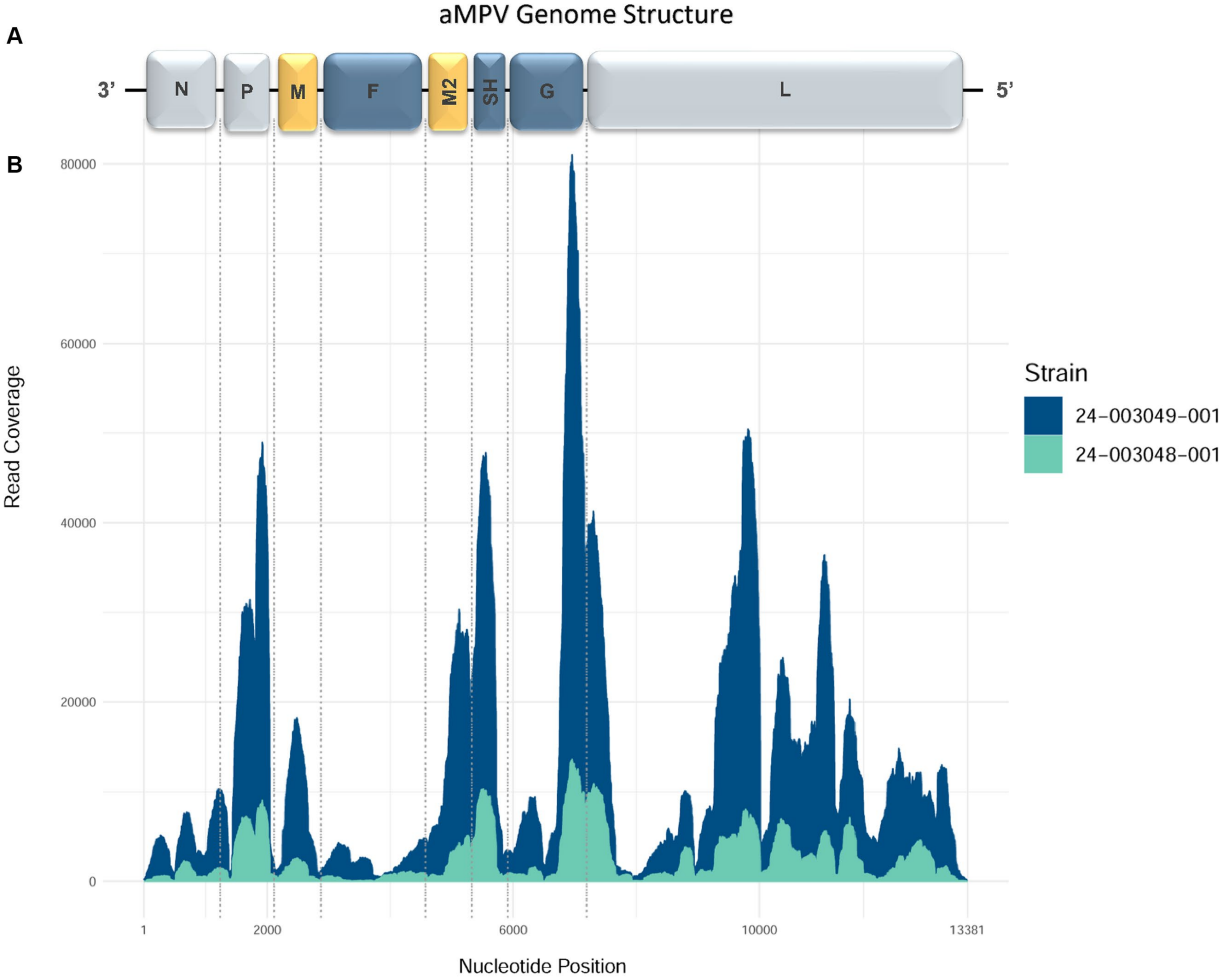
Genome organization and coverage of the aMPVs from this study. **(A)** Map of genomic RNAs in coding 3′-to-5′ orientation in which each box, drawn approximately to scale, represents a gene. Three nucleocapsid-associated proteins, namely a nucleoprotein (N), a phosphoprotein (P), and a large polymerase protein (L), are shown in grey; matrix proteins (M and M2) are shown in yellow; three glycosylated transmembrane surface envelope proteins, namely a fusion protein (F), an attachment protein (G), and a small hydrophobic protein (SH) are shown in blue. **(B)** Coverage plot showing a number of reads aligning to the consensus sequence (y-axis) along the length of the consensus sequence (x-axis, length in nucleotides, corresponding to the diagram of the viral genome above).

The MiSeq run generated 5,203,334 to 6,087,628 total raw paired-end reads per sample (Table 3). *De novo* assembly resulted in one complete genome sequence (100% genome breadth coverage) and one near-complete genome sequences (99.42% genome breadth coverage) of aMPVs, with a mean read depth of 17,358 and 2,732, respectively (Figure 1). These genome sequences were designated as turkey/US/CA/24–003048-001/2024 and turkey/US/CA/24–003049-001/2024 and have been deposited in GenBank under accession numbers PP442011.1 and PP442012.1, respectively.

**Table 3:** Summary of aMPV-A sequence assembly.

Sample pool	Total raw reads	aMPV reads	Mean depth coverage, reads	Genome breadth coverage, %	GenBank accession number
24–003038-001	5,203,334	17,882	195	71.86	N/A
24–003047-001	6,087,628	1,013	11	27.70	N/A
24–003048-001	5,089,336	219,102	2,732	99.42	PP442011
24–003049-001	5,562,008	987,843	17,358	100	PP442012

**Figure 2:**
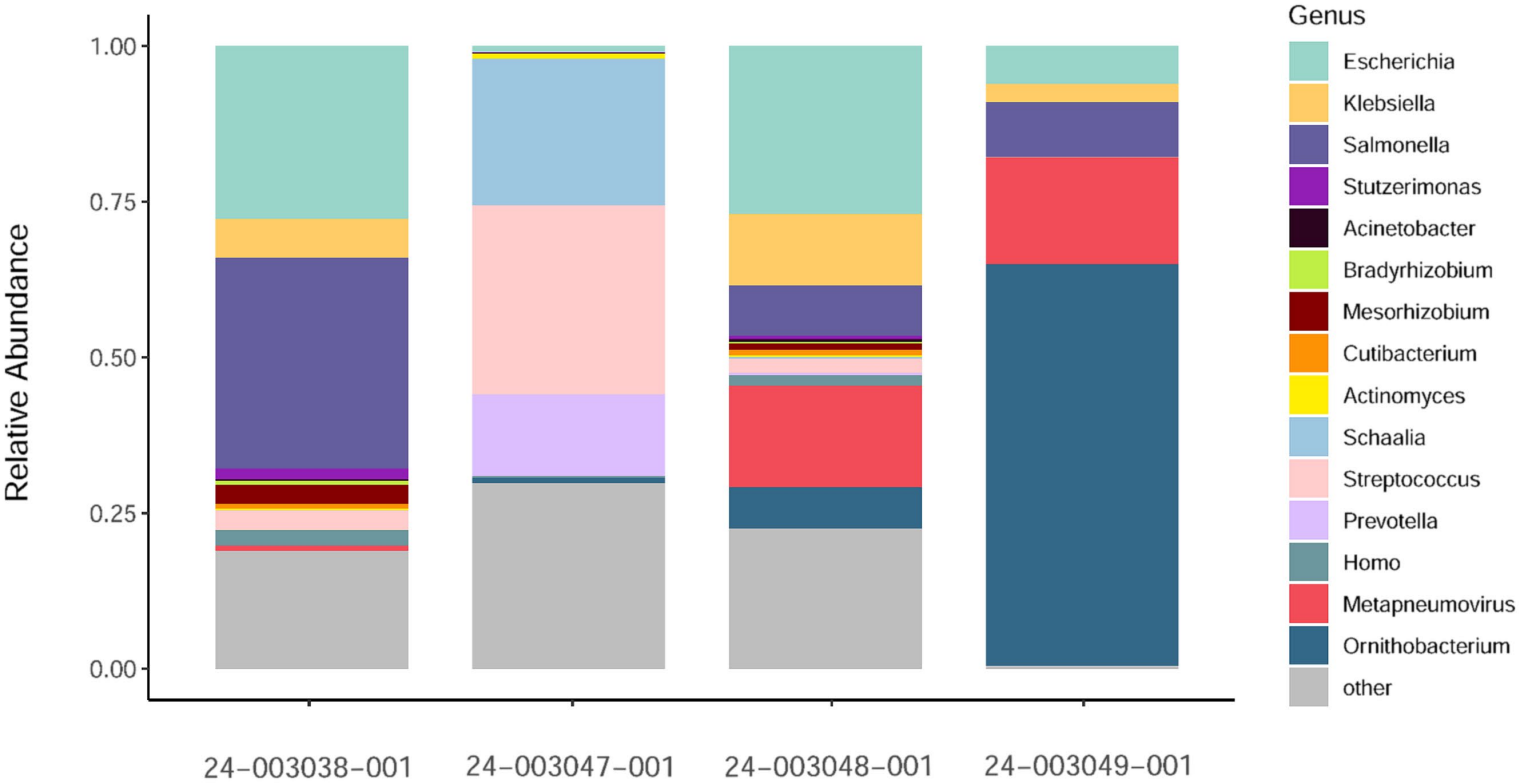
Relative abundance of non-host reads at the genus level as estimated by Kraken2/Bracken obtained. Only a subsample of 15 taxa with the greatest median relative abundances from the total community is shown.

Additionally, we investigated the presence of possible co-infections by assessing species abundance estimates using Kraken2/Bracken analysis in the sequenced samples (Figure 2). Samples 24–003048-001 and 24–003049-001, which exhibited the highest number of aMPV reads, showed a similar pattern of microbial relative abundance with high levels of reads from the *Escherichia*, *Klebsiella*, *Salmonella*, and *Ornithobacterium* genera. Sample 24–003038-001 showed a higher abundance of reads from the *Salmonella* genus and lacked reads from the *Ornithobacterium* genus. Sample 24–003047-001 exhibited Ornithobacteriun reads, but lacked reads from the *Klebsiella* and *Salmonella* genera, instead showing a high abundance of reads from the *Schaalia, Streptococcus*, and *Prevotella* genera.

### 3.3 Molecular characterization

The complete genome sequence of turkey/US/CA/24–003049-001/2024 was 13,381 nucleotides (nt) long. Both obtained genome sequences are consistent with the organization and sequence lengths of previously reported aMPVs (Rima et al., 2017). Their genomes comprised eight known aMPV genes encoding nine proteins: N (1,176 nt), P (837 nt), M (765 nt), F (1,617 nt), M2-1 (561 nt), M2-2 (222 nt), SH (525 nt), G (1,176 nt), and L (6,015 nt), which are further flanked by a leader sequence at 3′ end and trailer sequence at the 5′ end. The 3′-leader and 5′-trailer genomic regions of 24–003048-001 sequence lacked read coverage. However, based on 24–003049-001 complete genome sequence, similar to Mexican aMPVs collected between 2020 and 2022 (Kariithi et al., 2022), the 3′-leader is 55 nt-long and the 5′-trailer is 115 nt-long. The numbers of nucleotides in the non-coding intergenic sequences (IGS) of all three aMPVs were consistent with other aMPVs: IGS between N/P genes (*n* = 24 nt), P/M (*n* = 25 nt), M/F (*n* = 67 nt), F/M2 (*n* = 26 nt), M2-1/M2-2 overlap (*n* = 44 nt) M2/SH (*n* = 52 nt), SH/G (*n* = 82 nt), and G/L (*n* = 85 nt). Both the organization and length of these genome sequences were similar to previously reported Mexican aMPV-A strains (Kariithi et al., 2022).

The genome sequences of 24–003048-001 and 24–003049-001 US aMPVs shared 99.83% nucleotide identity. BLAST comparison to the currently published aMPV showed that both sequences were most closely related to four Mexican aMPV-A strains: 3155/22 (99.47 and 99.64%) (GenBank accession number ON854014.1), 2518/22 (99.08 and 99.24%) (GenBank accession number ON854006.1), 3153/22 (98.99 and 99.16%) (GenBank accession number ON854012.1), and 3154/22 (99.0 and 99.17%) (GenBank accession number ON854013.1) and showed the lowest identities to the Brazilian strain BR-SP/669/03 (96.86 and 97.02%) (GenBank accession number MF093139.1). The G gene sequences of the US isolates were identical and were most similar to strain 3155/22 at both the nucleotide (99.15%) and amino acid (97.95%) levels, and least similar to German strains 755/08 and 1133/07 (93.16%) at the nucleotide level (GenBank accession number JF793650.1 and JF793651.1, respectively) and to German strain 761/88 (88.42%) (GenBank accession number JF793655.1) at the amino acid level.

Detailed amino acid analysis revealed that both US aMPVs had the same 91 non-synonymous mutations as the closest Mexican strains 3155/22 when compared to the reference aMPV-A strain LAH A (GeneBank accession number NC_039231.1) (Supplementary Table S1). Additionally, the US aMPVs exhibited 9 non-synonymous mutations that were not observed in the Mexican 3155/22 or reference LAH A sequences. The majority of these mutations predictably occurred in the most diverse G gene.

### 3.4 Phylogenetic analysis

**Figure 3:**
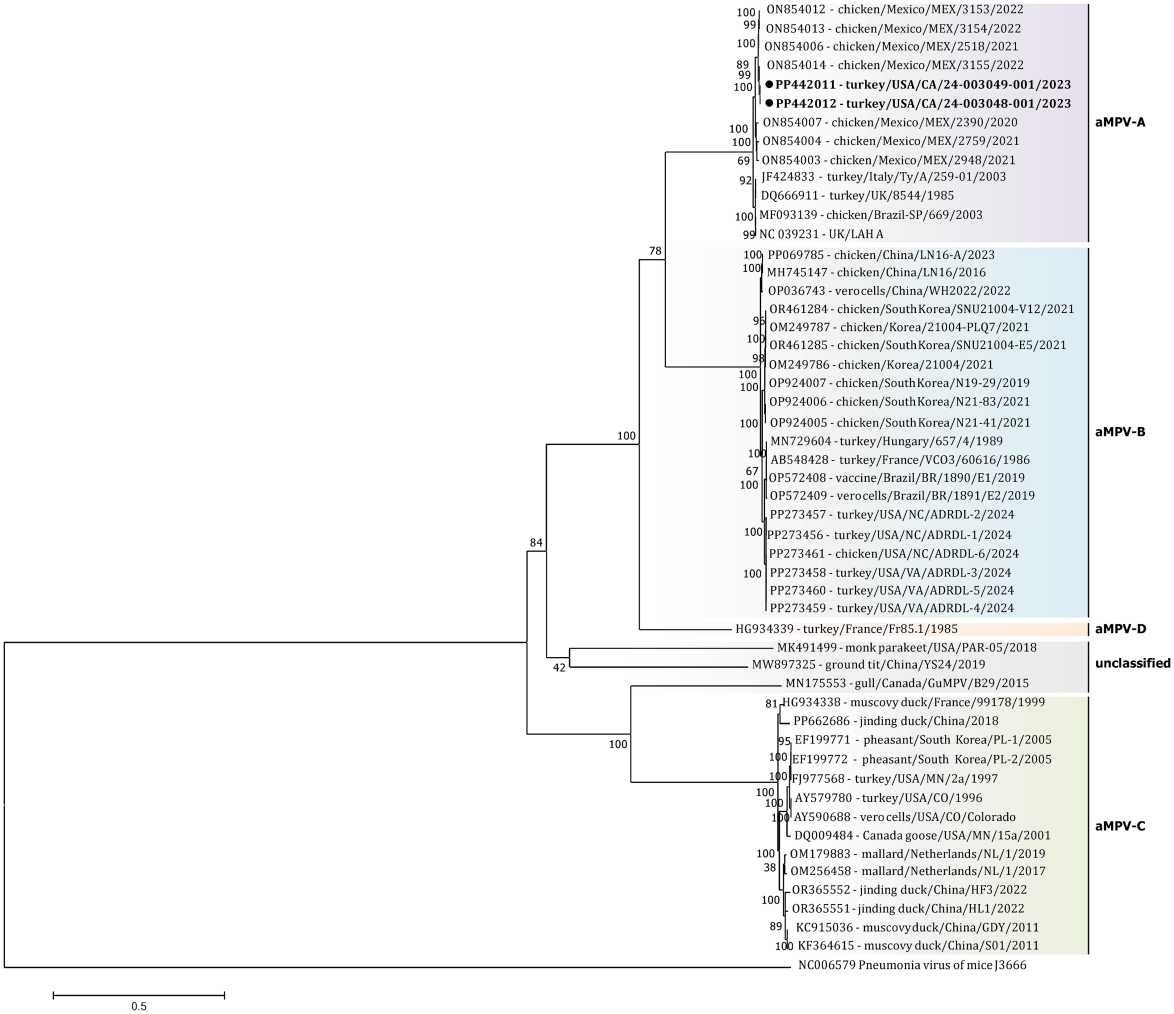
Phylogenetic analysis of US aMPV-A strains 24–003048-001 and 24–003049-001 (marked with a black circle) within the genus *Metapneumovirus* based on complete nucleotide genome sequences. Strain names include GenBank accession numbers, along with host species, country origin, strain name, and year of sample collection. The tree was constructed using the Maximimum-Likelihood method based on the best-fitting General-Time Reversible (GTR + G + I) substitution model with 1,000 bootstraps. The tree is drawn to scale, with branch lengths measured in the number of substitutions per site. The analysis involved 52 nucleotide sequences (sequence from *Murine* orthopneumovirus (NC_006579) was included as an outgroup). All positions containing gaps and missing data were eliminated. There was a total of 10,787 positions in the final data set.

The initial phylogenetic tree constructed based on all available complete and near-complete nucleotide sequences of all aMPV subtypes confirmed the clustering of both US strains within aMPVs of A subtype. Interestingly, phylogenetic analysis revealed that aMPV-A strains were split into two distinct clusters, with the US strains from this study clustering together with the Mexican strains (Figure 3).

**Figure 4:**
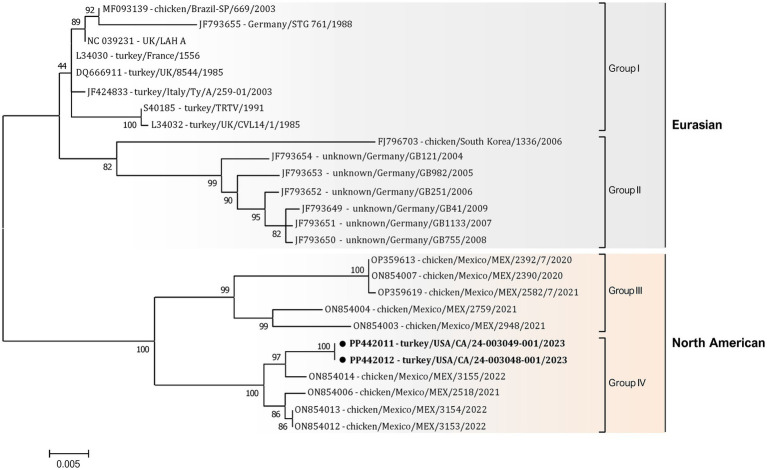
Phylogenetic analysis of US aMPV-A strains 24-003048-001 and 24-003049-001 (marked with a black circle) within the aMPV-A strains based on complete nucleotide G gene sequences. Strain names include GenBank accession numbers, along with host species, country origin, strain name, and year of sample collection. The tree was constructed using the Maximum-Likelihood method based on the best-fitting Tamura-Nei (TN93 + G) substitution model with 1,000 bootstraps. The tree is drawn to scale, with branch lengths measured in the number of substitutions per site, and the tree is midpoint rooted. The analysis involved 26 nucleotide sequences. All positions containing gaps and missing data were eliminated. There was a total of 1,141 positions in the final data set.

Additionally, a detailed phylogenetic analysis was performed based on all available near-complete G gene nucleotide sequences (*n* = 26), the most variable gene of all metapneumovirus (Figure 4). The phylogenetic analysis showed the split of currently available aMPV-A strains into two distinct clusters: an older Eurasian cluster (late 1980s–early 2000s) and a more recent North American (2020–2023) (Figure 4). Based on G gene nucleotide sequences, aMPV-A strains from those two clusters had a between-group mean *p*-distance of 0.058 and within-group mean *p*-distance of 0.024 and 0.028 in the Eurasian and North American clusters, respectively. The US strains from this study clustered together with the Mexican strains, further expanding the North American cluster of aMPV-As. Specifically, both strains from this study were closely related to strain 3155/22 (GenBank accession number ON854014.1) among other Mexican strains. Interestingly, all Mexican strains were derived from chickens, unlike the US strains from turkeys, and were further split into two groups with a between-group *p*-distance of 0.031. The US strains from turkeys, together with the Mexican strains from chickens from group IV, were more closely related with a within-group *p*-distance of 0.006 as compared to the rest of the Mexican strains from group III, which had a within-group *p*-distance of 0.029.

### 3.5 RT-qPCR test comparison

**Figure 5:**
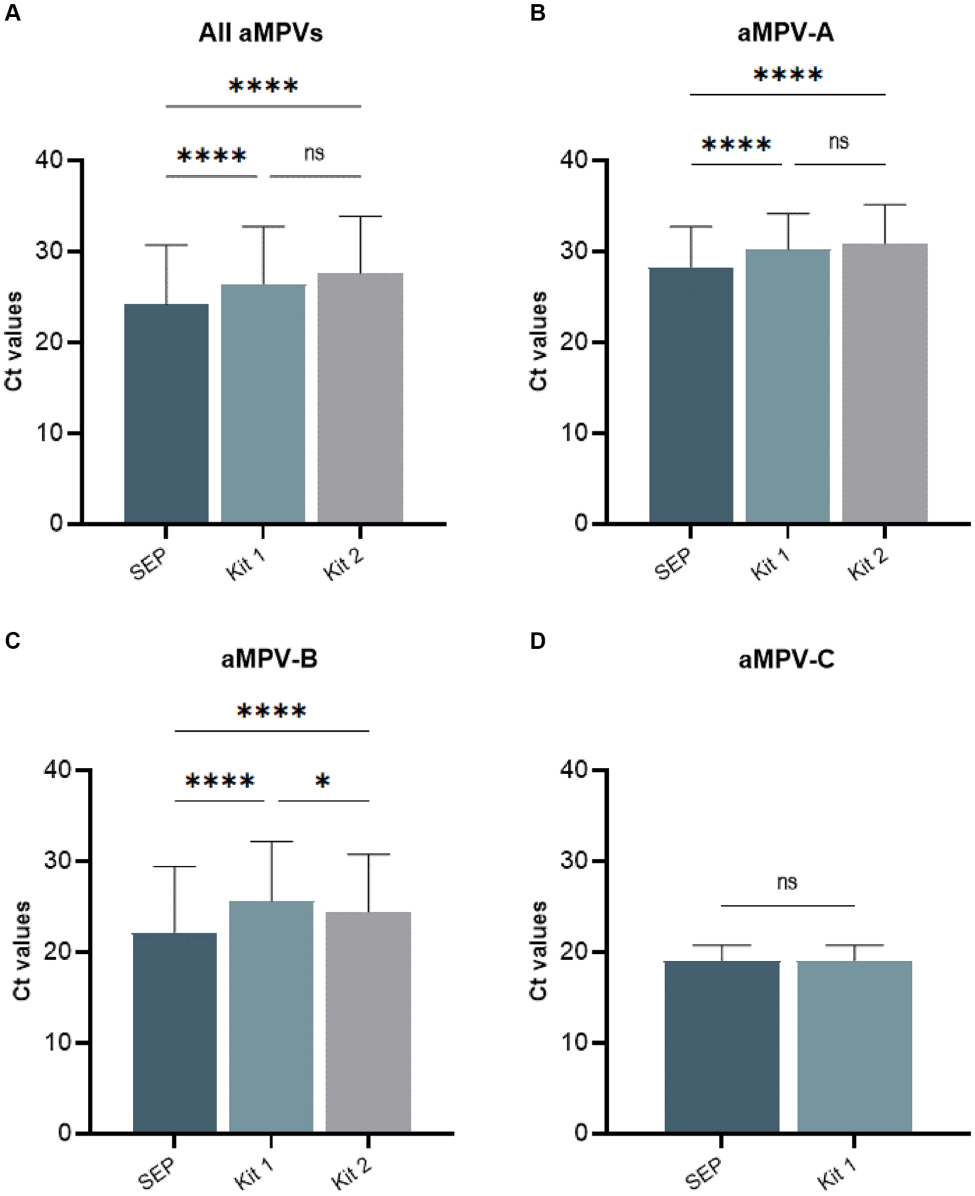
Comparison of average cycle threshold (Ct) values of SEP, Kit 1, and Kit 2 RT-qPCR tests on all tested aMPV samples **(A)**, subtype A **(B)**, subtype B **(C)**, and subtype C **(D)**.

Our previously published SEP aMPV RT-qPCR test (Kariithi et al., 2022) was revised to include the detection and subtyping of aMPV-B and -C as well. To achieve this, the aMPV-B primers/probe set from the study by Guionie et al. (2007) were modified to be more conserved based on SNP analysis using currently available aMPV sequences. Additionally, aMPV-C primers/probe set from Jardine et al. (2018) study were incorporated into to SEP aMPV subtype specific RT-qPCR test. The performance of our revised test was evaluated by comparison with the two European commercial kits. Kit 1 was designed to detect and differentiate three aMPV subtypes (A, B, and C) in different reactions, while Kit 2 differentiated only the A and B subtypes. However, it could perform detection simultaneously in a one-tube reaction with shared reagents and a single real-time PCR program. The results, summarized in Table 4, demonstrated that all three tests detected their target subtype (*n* = 36) in 100% of known positive samples. While overall commercial kits showed no statistically significant difference between their Ct values, SEP test provided significantly lower Ct values (*p* < 0.0001) (Figure 5A). Specifically, SEP test detected aMPV-A and -B at lower Ct values (*p* < 0.0001) compared to Kit 1 and Kit 2 (Figures 5B,C), while no difference was observed in aMPV-C detection between SEP test and Kit 1 (Figure 5D).

**Table 4:** Subtype identification and cross-reactivity comparison of SEP aMPV subtype-specific RT-qPCR test with commercial kits.

Sample	Subtype	aMPV-A, Ct^a^			aMPV-B, Ct			aMPV-C, Ct		
		SEP^b^	Kit 1^c^	Kit 2^d^	SEP	Kit 1	Kit 2	SEP	Kit 1	Kit 2
MEX/1863/2019	A	24.9	27.5	27.0	–	–	26.2	–	–	n/a^e^
MEX/1877/2019		–^f^	38.8	38.8	–	–	29.0	–	–	n/a
MEX/1878/2019		31.5	35.0	37.3	–	–	33.1	–	–	n/a
MEX/2390/2020		25.6	27.6	28.5	–	–	30.3	–	–	n/a
MEX/2392/2020		24.8	27.2	28.1	–	–	32.2	–	–	n/a
MEX/2948/2021		29.6	29.9	30.6	–	–	30.9	–	–	n/a
MEX/2721/2021		34.7	31.7	31.8	–	–	33.2	–	–	n/a
MEX/2582/2021		29.2	26.7	27.3	–	–	32.4	–	–	n/a
MEX/2566/2021		35.6	32.2	31.1	–	–	29.8	–	–	n/a
MEX/2939/2021		31.6	26.5	26.5	–	–	33.8	–	–	n/a
US/CA/24–003038-001/2023		31.1	33.1	35.1	–	33.9	–	–	–	n/a
US/CA/24–003038-001/2023		32.4	35.2	37.1	–	–	–	–	–	n/a
US/CA/24–003038-001/2023		26.3	28.7	30.2	–	–	–	–	–	n/a
US/CA/24–003038-001/2023		23.2	25.6	27.4	–	–	–	–	–	n/a
UK/CVL14-1/1985		30.6	28.2	26.7	–	37.6	36.2	–	–	n/a
US/GA/11281543/2024	B	–	–	–	20.2	24.0	22.8	–	–	n/a
US/GA/11281545_2/2024		–	–	–	16.0	20.0	19.1	–	–	n/a
US/GA/11283628/2024		–	–	–	17.4	21.0	20.0	–	–	n/a
US/GA/11283629/2024		–	–	–	21.0	24.9	23.6	–	–	n/a
US/GA/11283951/2024		–	–	–	20.6	24.4	23.1	–	–	n/a
US/GA/11283954_H1A/2024		–	–	–	19.2	22.8	21.6	–	–	n/a
US/GA/11284245_dead/2024		–	–	–	15.7	19.3	18.4	–	–	n/a
US/GA/11284246/2024		–	–	–	16.7	20.3	19.3	–	–	n/a
US/GA/11284269_1/2024		–	–	–	15.4	18.9	18.0	–	–	n/a
US/GA/11284396/2024		–	–	–	15.5	19.1	18.1	–	–	n/a
US/NC/126/2024		–	–	–	21.8	28.4	28.4	–	–	n/a
US/NC/1124/2024		–	–	–	32.0	35.3	33.9	–	–	n/a
USA/NC/1724/2024		–	–	–	33.4	36.5	35.3	–	–	n/a
US/NC/9123/2023		–	–	–	30.8	34.2	32.5	–	–	n/a
Hungary/657/4/1989		–	–	–	36.5	35.2	32.6	–	–	n/a
US/MN/1a/1997	C	–	–	–	–	–	–	20.2	19.8	n/a
US/CO/97/1997		–	–	–	–	–	–	18.3	18.3	n/a
US/MN/2a/1997		–	–	–	–	–	–	16.2	16.2	n/a
US/CO-01/1997		–	–	–	–	–	–	20.7	20.9	n/a
US/MN/1b/1997		–	–	–	–	–	–	20.2	20.1	n/a
US/CO-02/1997		–-	–	–	–	–	–	18.7	19.2	n/a

a Ct values, RT-qPCR cycle threshold values.

b SEP, In house SEP aMPV subtype-specific RT-qPCR test.

c Kit 1, VetMax Avian Metapneumovirus Kit (Thermo Fisher Scientific, France).

d Kit 2, RealPCR AMPV A/B Multiplex RNA Mix (IDEXX, France).

e n/a, not applicable.

f no Ct value could be determined at the end of the 40 RT-qPCR cycles.

Additionally, we conducted cross-detection testing by analyzing aMPV samples of known subtypes with settings intended for other subtypes. While no false-positives were observed with aMPV-B and -C, Kit 1 and Kit 2 yielded false-positive results for aMPV-A when tested with subtype B settings (Table 4). The SEP kit did not exhibit any false-positive results.

Based on the Ct values of the 10-fold diluted RNAs, similar results were obtained with the SEP test and the commercial kits, where the end-point detection was at the same dilution, albeit with slightly lower Ct (Supplementary Table S2). This ensures reliable detection of samples with lower titers.

## 4 Discussion

The recent emergence of aMPV subtypes A and B in the United States poultry industry, following a prolonged absence of aMPV detection since the eradication of subtype C in the early 2000s, highlights the challenges in preventing its spread despite stringent biosecurity measures. Our study presents the first detection and molecular characterization of aMPV-A in the US, emphasizing the importance of vigilant surveillance and diagnostic capabilities in detecting and managing emerging viral threats.

Historically, aMPV of subtype A was predominantly detected in high-density poultry farm areas in European countries, South America (Brazil), and Africa (Nigeria, Egypt, and South Africa), with sporadic reports from North America (Mexico) and Asia (China and South Korea) (Umar et al., 2019; Salles et al., 2023). However, recent trends indicate a decline in the detection of subtype A and an increase in the prevalence of subtype B in these regions. The shift in the predominance of aMPV subtypes from A to B and increased detection of aMPV-A in North America observed in recent years is noteworthy and may reflect ongoing evolutionary dynamics and changing epidemiological patterns. Several factors may contribute to this observed shift in geographical subtype prevalence, including evolutionary pressures, such as host immunity, viral adaptation, and environmental factors, that may influence the fitness and transmission dynamics of different aMPV subtypes. Changes in poultry production practices, trade patterns, and international travel may also contribute to the dissemination of specific viral strains and the emergence of new subtypes in different geographic regions. It bears noting the recent introduction of aMPV-A and -B into the US has coincided with increased spread of H5N1 HPAI by migratory waterfowl over the past several years. Whether these two occurrences are causal relationships or random effects remains to be determined.

The molecular characterization, including complete genome sequencing and phylogenetic analysis, of aMPV subtype A genomes from the US provided valuable insights into the genetic diversity and evolution of aMPVs. Phylogenetic analysis revealed close genetic relatedness between the US isolates collected from turkeys in 2023 and Mexican strains from chickens in 2020–2022 (Kariithi et al., 2022), suggesting potential transboundary spread of aMPV strains. Notably, the US and Mexican aMPV-A strains were grouped into a distinct cluster separate from Eurasian aMPV-A strains based on alignments of the currently available complete genomes and G genes, that is used in metapneumovirus classification. The close evolutionary connection between the identified US and Mexican aMPV-A strains suggests an epidemiologic connection, but it is unclear if the virus was introduced directly by poultry or poultry products or potentially transmitted through wild birds (Wille et al., 2017; Graziosi et al., 2022). Furthermore, the detailed amino acid analysis identified additional mutations in the G gene of US isolates compared to the Mexican strains, potentially indicating ongoing viral evolution or adaptation within the US turkey population. Although it remains to be experimentally demonstrated whether these genetic variations affect pathogenicity, phylogenetic clustering, host adaptation, or the emergence and circulation of a unique North American group of aMPV-As. Additionally, our study demonstrated the presence of co-infection with potential bacterial pathogens from *Escherichia*, *Klebsiella*, *Prevotella, Salmonella*, *Schaalia, Streptococcus*, and *Ornitobacterium* genera in aMPV-A-positive samples. These findings are in accordance with other studies suggesting that co-infection with other pathogens, such as *Escherichia* (Turpin et al., 2002; Salles et al., 2023), *Mycoplasma gallisepticum* (Naylor et al., 1992), *Ornithobacterium rhinotracheale* (Marien et al., 2005; Zuo et al., 2018), or lentogenic Newcastle disease virus (Turpin et al., 2002), may exacerbate clinical signs, increase viral shedding, and prolong shedding duration. However, the limitation of this research is that the co-infections detected in the pooled swabs could result from aMPV-uninfected samples, necessitating further studies to confirm these findings.

The sequence analysis presented in this study does not provide definitive insights into the pathogenicity of the newly identified aMPV-A strains, as no specific molecular markers for pathogenicity are recognized. Despite the detection of these aMPV-A sequences in apparently sick turkeys, it is challenging to predict the pathogenic potential of the newly identified strains for turkeys or other avian species. Previous experimental studies have demonstrated that all subtypes of aMPV are capable of infecting birds from the order *Galliformes*, although variations exist in susceptibility, clinical manifestation development, and shedding patterns (Brown et al., 2019; Tucciarone et al., 2022). Turkeys have shown susceptibility to and the ability to transmit all four subtypes, except for subtype aMPV-C of duck lineage. Chickens exhibited full susceptibility to subtype B, with seroconversion occurring in the absence of shedding for subtype A, subtype C of turkey lineage, and subtype C of duck lineage with an absence of clinical signs (Brown et al., 2019). Ducks supported viral replication and displayed clinical signs only upon challenge with subtype aMPV-C of duck lineage.

Overall, the susceptibility of galliformes to different aMPV subtypes together with a shift in subtype predominance, detection of two new aMPV subtypes in North America (Canuti et al., 2019; Retallack et al., 2019), and the emergence of aMPV-A and -B subtypes in the United States (Luqman et al., 2024; UGA, 2024; United Egg Producers, 2024) underscores the importance of continuous surveillance, diagnostics, and biosecurity measures in mitigating the impact of aMPV infections on poultry health and production. Our study demonstrates the utility of molecular diagnostic methods, such as RT-qPCR, in detecting and subtyping aMPV strains with high sensitivity and specificity. The SEP aMPV subtype-specific RT-qPCR test demonstrated similar or better performance compared to European commercial kits. The SEP RT-qPCR tests were modified from earlier published studies (Guionie et al., 2007; Jardine et al., 2018; Kariithi et al., 2022) and the primers were slightly modified to be more conserved to available sequences in GenBank based on SNP analysis (Ferreira and Suarez, 2019). The protocols were also modified using reagents commonly used in US NAHLN labs with standardized cycling conditions so that A, B, and C subtypes could be evaluated at one time, although as three separate reactions. All three methods of aMPV detection were felt to be adequate for detection of the most common subtypes, and the choice of which test to use is often dependent on cost, usage, availability, and number of samples tested annually. These findings highlight the importance of updating RT-qPCR primers and probes to align with the genetic diversity of currently circulating aMPV isolates, ensuring accurate and reliable detection. The efficacy and reliability of SEP aMPV subtype-specific RT-qPCR demonstrated its suitability as an alternative while commercial kits undergo CVB licensure to become available on the US market.

In conclusion, the molecular characterization based on the obtained complete genome of the first aMPV subtype A detected in the US provides valuable insights into the epidemiology, genetic diversity, and diagnostic methods of this economically significant poultry pathogen. Continued surveillance, research, and collaboration are essential for effective disease management and control to safeguard poultry health and ensure the sustainability of the poultry industry in the face of emerging viral threats.

## 5 Conclusion

This study presents the first detection of aMPV subtype A in the United States. Through the molecular characterization of the complete aMPV subtype A genome, we have provided novel insights into the genetic diversity and evolutionary dynamics of aMPVs, highlighting their potential for transboundary spread and impact on poultry health. Furthermore, the performance of our revised aMPV subtype-specific RT-qPCR test, alongside commercial kits, demonstrated value in detecting and subtyping aMPV strains with high sensitivity and specificity. Additionally, the absence of cross-reactivity observed in our test highlights its utility in reliably differentiating aMPV subtypes, facilitating targeted disease management strategies. Overall, our findings underscore the importance of proactive measures in the face of the recent detection of subtypes A and B in the US after a prolonged period without aMPV detection in the country. This emphasizes the need for continued surveillance to monitor the emergence of aMPV and implement effective biosecurity protocols to mitigate the impact of aMPV on poultry health and industry sustainability.

## Data availability statement

Genomic sequence data presented in this study have been deposited in GenBank under accession numbers: PP442011 and PP442012.

## Ethic statement

Ethics approval and specific consent procedures were not required for this study. Samples from this study were voluntarily submitted to the USDA’s National Veterinary Services Laboratories as part of routine surveillance and support of the national flock. The USDA further analyzed and collated these data under the authority of the Animal Health Protection Act.

## Author contributions

IG: Conceptualization, Data curation, Formal analysis, Investigation, Methodology, Software, Validation, Visualization, Writing – original draft. MT: Formal analysis, Investigation, Methodology, Software, Validation, Writing – review & editing. MK: Formal analysis, Investigation, Methodology, Software, Validation, Writing – review & editing. DK: Funding acquisition, Methodology, Resources, Supervision, Writing – review & editing. KS: Methodology, Resources, Writing – review & editing. AK: Methodology, Resources, Writing – review & editing. DS: Conceptualization, Data curation, Formal analysis, Funding acquisition, Methodology, Project administration, Supervision, Writing – review & editing.

## Acknowledgments

The authors thank Ricky Zoller and Kerrie Franzen for their technical assistance with this work.
