# Supplementary material for: Introduction of Avian metapneumovirus subtype A to the United States: molecular insights and implications
Source: Front Microbiol. 2024 Jul 5;15:1428248. doi: 10.3389/fmicb.2024.1428248 (PMC11258015; doi:10.3389/fmicb.2024.1428248)
Supplement: Supplementary file 1 [file Table_1.DOCX]

Supplementary Table 1. Non-synonymous mutations of US aMPV-A strain 24-003049-001 compared to the reference strain and the most closely related Mexican strain.

| Gene |  | Reference LAH A | Mexican 3155/22 |  | Gene | Reference LAH A | Mexican 3155/22 |
| --- | --- | --- | --- | --- | --- | --- | --- |
| N |  | T46I |  |  | G | S117N |  |
|  |  | S101P |  |  |  | D120N |  |
|  |  | S105P |  |  |  | V133A |  |
|  |  | T129A |  |  |  | A151V |  |
|  |  | D139E |  |  |  | **D158G** | **D158G** |
|  |  | R356K |  |  |  | S175N |  |
| P |  | K61R |  |  |  | **P180L** | **P180L** |
|  |  | P73S |  |  |  | A186T |  |
|  |  | E104K |  |  |  | P188H |  |
|  |  | I245V |  |  |  | D196N |  |
|  |  | E265D |  |  |  | G201S |  |
|  |  | **S266F** | **S266F** |  |  | S213G |  |
|  |  | D267N |  |  |  | P230S |  |
| M |  | N171D |  |  |  | G240R |  |
| F |  |  | I3V |  |  | T258N |  |
|  |  | S16G |  |  |  | G260V |  |
|  |  | N54S |  |  |  | M269T |  |
|  |  | Q179R |  |  |  | L271P |  |
|  |  | R233K |  |  |  | P272L |  |
|  |  | **A249S** | **A249S** |  |  | S274G |  |
|  |  | K294E |  |  |  | D276N |  |
|  |  | R295E |  |  |  | **P287L** | **P287L** |
|  |  | R2296K |  |  |  | F292L |  |
|  |  | K323E |  |  |  |  | H295Y |
|  |  | L345S |  |  |  | P301L |  |
|  |  | D348E |  |  |  | **S308P** | **S308P** |
|  |  | S387G |  |  |  | P310S |  |
|  |  | V453E |  |  |  | D322N |  |
|  |  | L506V |  |  |  | K326R |  |
|  |  | S514F |  |  |  | Y368N |  |
| M2-1 |  | L47V |  |  | L | Y44H |  |
|  |  | **A93S** | **A93S** |  |  | R133C |  |
|  |  | S142N |  |  |  | R313Q |  |
|  |  | P169S |  |  |  | G328S |  |
| M2-2 |  | R8K |  |  |  | A817S |  |
|  |  |  | R26K |  |  | V862I |  |
|  |  | V46I |  |  |  | D877N |  |
|  |  | L63F |  |  |  | I1103M |  |
| SH |  | T16K |  |  |  | A1204V |  |
|  |  | V62I |  |  |  | L1335I |  |
|  |  | R102G |  |  |  | H1354R |  |
|  |  | P106L |  |  |  | P1397L |  |
|  |  | N174I |  |  |  |  | I1423V |
| G |  | D23N |  |  |  | T1557N |  |
|  |  |  | K77E |  |  | S1739G |  |
|  |  | P79S |  |  |  |  | G1916D |
|  |  | A88T |  |  |  | **K1950R** | **K1950R** |
|  |  |  | R93G |  |  | N1986T |  |
|  |  | **E103G** | **E103G** |  |  | V1986I |  |
