# Supplementary material for: Introduction of Avian metapneumovirus subtype A to the United States: molecular insights and implications
Source: Front Microbiol. 2024 Jul 5;15:1428248. doi: 10.3389/fmicb.2024.1428248 (PMC11258015; doi:10.3389/fmicb.2024.1428248)
Supplement: Supplementary file 2 [file Table_2.DOCX]

**Supplementary Table 2. Comparison of the limit of detection of SEP aMPV subtype-specific RT-qPCR test with commercial kits.**

| **aMPV-A** | | | | |  | **aMPV-B** | | | | | | | | |  | | | **aMPV-C** | | | | | | | | |  |
| --- | --- | --- | --- | --- | --- | --- | --- | --- | --- | --- | --- | --- | --- | --- | --- | --- | --- | --- | --- | --- | --- | --- | --- | --- | --- | --- | --- |
| **Sample** | **Dilutions** | **SEP^1^** | **Kit 1^2^** | **Kit 2^3^** |  | **Sample** | **Dilutions** | | **SEP** | | **Kit 1** | | **Kit 2** | |  | | **Sample** | | **Dilutions** | | **SEP** | | **Kit 1** | | **Kit 2** | |  |
| **UK/1411** | 10^-1^ | 25.6 | 28.2 | 26.7 |  | **US/GA/11284269/2024** | | 10^-1^ | | 17.3 | | 19.5 | | 18.8 | |  | | **US/MN/1a/1997** | | 10^-2^ | | 22.9 | | 23.6 | | n/a^4^ | |
|  | 10^-2^ | 29.9 | 31.4 | 30.3 |  |  |  | 10^-2^ | | 20.7 | | 22.9 | | 22.0 | |  | |  |  | 10^-3^ | | 26.6 | | 27.1 | | n/a | |
|  | 10^-3^ | 33.9 | 34.2 | 33.8 |  |  |  | 10^-3^ | | 23.9 | | 26.5 | | 25.5 | |  | |  |  | 10^-4^ | | 29.9 | | 30.7 | | n/a | |
|  | 10^-4^ | 35.7 | -^5^ | 37.2 |  |  |  | 10^-4^ | | 27.3 | | 29.9 | | 28.8 | |  | |  |  | 10^-5^ | | 32.8 | | 34.1 | | n/a | |
|  | 10^-5^ | - | - | - |  |  |  | 10^-5^ | | 30.8 | | 32.9 | | 32.3 | |  | |  |  | 10^-6^ | | - | | - | | n/a | |
|  | 10^-6^ | - | - | - |  |  |  | 10^-6^ | | 33.2 | | 36.0 | | 37.5 | |  | |  |  | 10^-7^ | | - | | - | | n/a | |
|  | 10^-7^ | - | - | - |  |  |  | 10^-7^ | | 35.4 | | 38.3 | | 36.4 | |  | |  |  | 10^-8^ | | - | | - | | n/a | |
|  | 10^-8^ | - | - | - |  |  |  | 10^-8^ | | - | | - | | - | |  | |  |  | 10^-9^ | | - | | - | | n/a | |
| **MEX/2582/2021** | 10^-1^ | 23.7 | 26.7 | 27.3 |  | **US/GA/11284396/2024** | | 10^-1^ | | 17.6 | | 19.9 | | 18.9 | |  | | **APV-CO-01** | | 10^-2^ | | 23.7 | | 24.8 | | n/a | |
|  | 10^-2^ | 28.4 | 30.1 | 30.2 |  |  |  | 10^-2^ | | 21.0 | | 23.3 | | 22.4 | |  | |  |  | 10^-3^ | | 26.7 | | 28.2 | | n/a | |
|  | 10^-3^ | 31.6 | 33.7 | 33.5 |  |  |  | 10^-3^ | | 24.3 | | 26.8 | | 25.6 | |  | |  |  | 10^-4^ | | 30.3 | | 31.8 | | n/a | |
|  | 10^-4^ | 35.3 | 37.3 | 37.2 |  |  |  | 10^-4^ | | 27.8 | | 30.5 | | 29.2 | |  | |  |  | 10^-5^ | | 33.1 | | 34.3 | | n/a | |
|  | 10^-5^ | - | - | - |  |  |  | 10^-5^ | | 30.9 | | 33.9 | | 32.3 | |  | |  |  | 10^-6^ | | - | | - | | n/a | |
|  | 10^-6^ | - | - | - |  |  |  | 10^-6^ | | 33.6 | | 38.4 | | 35.8 | |  | |  |  | 10^-7^ | | - | | - | | n/a | |
|  | 10^-7^ | - | - | - |  |  |  | 10^-7^ | | - | | - | | - | |  | |  |  | 10^-8^ | | - | | - | | n/a | |
|  | 10^-8^ | - | - | - |  |  |  | 10^-8^ | | - | | - | | - | |  | |  |  | 10^-9^ | | - | | - | | n/a | |

^1^ SEP, In house SEP aMPV subtype-specific RT-qPCR test; ^2^ Kit 1, VetMax Avian Metapneumovirus Kit (Thermo Fisher Scientific, France); ^3^ Kit 2, RealPCR AMPV A/B Multiplex RNA Mix (IDEXX, France); ^4^ n/a, not applicable; ^5^ -, no Ct value could be determined at the end of the 40 RT-qPCR cycles.
